# Concurrent validity of barbell force measured from video-based barbell kinematics during the snatch in male elite weightlifters

**DOI:** 10.1371/journal.pone.0254705

**Published:** 2021-07-19

**Authors:** Ingo Sandau, Helmi Chaabene, Urs Granacher

**Affiliations:** 1 Research Group Weightlifting, Institute for Applied Training Science, Leipzig, Germany; 2 Division of Training and Movement Sciences, Research Focus Cognition Sciences, University of Potsdam, Potsdam, Germany; Universita degli Studi di Milano, ITALY

## Abstract

This study examined the concurrent validity of an inverse dynamic (force computed from barbell acceleration [reference method]) and a work-energy (force computed from work at the barbell [alternative method]) approach to measure the mean vertical barbell force during the snatch using kinematic data from video analysis. For this purpose, the acceleration phase of the snatch was analyzed in thirty male medal winners of the 2018 weightlifting World Championships (age: 25.2±3.1 years; body mass: 88.9±28.6 kg). Vertical barbell kinematics were measured using a custom-made 2D real-time video analysis software. Agreement between the two computational approaches was assessed using Bland-Altman analysis, Deming regression, and Pearson product-moment correlation. Further, principal component analysis in conjunction with multiple linear regression was used to assess whether individual differences related to the two approaches are due to the waveforms of the acceleration time-series data. Results indicated no mean difference (*p* > 0.05; *d* = −0.04) and an extremely large correlation (*r* = 0.99) between the two approaches. Despite the high agreement, the total error of individual differences was 8.2% (163.0 N). The individual differences can be explained by a multiple linear regression model (R^2^_adj_ = 0.86) on principal component scores from the principal component analysis of vertical barbell acceleration time-series waveforms. Findings from this study indicate that the individual errors of force measures can be associated with the inverse dynamic approach. This approach uses vertical barbell acceleration data from video analysis that is prone to error. Therefore, it is recommended to use the work-energy approach to compute mean vertical barbell force as this approach did not rely on vertical barbell acceleration.

## Introduction

Measuring force during resistance training exercises is important to assess and monitor training-induced responses of the neuromuscular system using for instance the force-velocity relationship [1, 2]. More specifically, during different drills such as the snatch, clean and jerk, squat jump, bench press throw, and bench press, the force that can be transferred from the lifter to the barbell is of major interest [3–6]. As it is difficult under real life conditions to directly measure force at the barbell (e.g., force transducer), barbell force has been determined indirectly using an inverse dynamic approach [4–6]. For this purpose, Newton’s second law of motion is used to compute barbell force from barbell mass and barbell acceleration. During weightlifting exercises, video-based barbell tracking is often applied to assess barbell acceleration, since this procedure can be used both during training and competition without affecting the lifters’ performance [7]. However, since video recorded barbell acceleration is the 2^nd^ derivative of barbell displacement, the signal is prone to measurement errors (i.e., measurement error from displacement increases during double-differentiation) [8]. Sato, Smith and Sands [9] analyzed the associations between vertical barbell acceleration during the performance of the high-pull exercise using video analysis and accelerometer data. For the two different assessment approaches, the reported correlation coefficients (i.e., cross-correlation) ranged between r = 0.95 to 0.99 for barbell acceleration time-series waveforms [9]. This non-perfect agreement of the vertical barbell acceleration time-series waveforms (video vs. accelerometer) is indicative of the error associated with the video-based barbell acceleration [10]. Therefore, assessing barbell force using inverse dynamics could be biased if video-based barbell acceleration is used as reference. For example, if the erroneously calculated barbell force is used to monitor training, incorrect conclusions may arise for training programming. Accordingly, a different methodological approach should be adopted to assess the barbell force using video analysis.

Mean vertical force can be assessed either through a work-energy approach or an inverse dynamic approach. Within the work-energy approach, mean vertical force is computed from change of kinetic energy (total work) using velocity, mass, and distance as input parameters [11]. Previous studies showed that this is a valid procedure to calculate mean vertical ground reaction force during the vertical jump [11] and vertical system force (barbell plus upper limbs) during the guided barbell bench press throw [12]. Although the work-energy approach seems applicable to measure mean vertical force during vertical jump and barbell bench throw, validity of the work-energy approach for the assessment of vertical barbell force is currently unresolved for weightlifting exercises.

Therefore, this study aimed to examine the agreement of computing mean vertical barbell force from video recordings using a work-energy approach (${\overset{-}{F}}_{\text{we}}$) compared with computing mean vertical barbell force using an inverse dynamic approach (${\overset{-}{F}}_{\text{id}}$) during the snatch exercise in male elite weightlifters. Further, as the acceleration-based error associated with ${\overset{-}{F}}_{\text{id}}$ is more prevalent during large and rapid changes of acceleration (e.g., around peak values) [10], we were interested in elucidating whether individual differences related to the two approaches are due to the waveforms of the acceleration time-series data. With reference to the work of Samozino et al. [11], we hypothesized that the work-energy approach represents a valid procedure to compute the mean vertical barbell force during the snatch in male elite weightlifters. Additionally, we expected that individual barbell acceleration time-series waveforms can explain individual differences between ${\overset{-}{F}}_{\text{we}}$ and ${\overset{-}{F}}_{\text{id}}$.

## Materials and methods

### Participants

Thirty male elite weightlifters with a mean age of 25.2±3.1 years (body mass: 88.9±28.6 kg) participated in this study. The weightlifters were the medal winners at the 2018 International Weightlifting Federation World Championships (Ashgabat, TKM) in the following 10 bodyweight categories (i.e., −55 kg, −61 kg, −67 kg, −73 kg, −81 kg, −89 kg, −96 kg, −102 kg, −109 kg, +109 kg). The study procedures were conducted with the approval of the International Weightlifting Federation and met the standards of the latest version of the Declaration of Helsinki. In addition, the experimental protocol was approved by the local Ethics Board of the Institute for Applied Training Science (No.: ER_2020.03.12_9). Due to the observational design of the study, informed consent was waived by the responsible Ethics Board.

### Data collection

All data were collected at the Senior World Championships 2018. For this study, the best successful snatch attempt from each athlete was selected for further analyses. The snatch lifts were video recorded and analyzed using the custom-made real-time barbell tracking software (Realanalyzer, IAT, Leipzig, Germany) [7]. The position of the digital camera (Canon, Legria HF G26) followed a routine set-up. The camera was placed at a distance of 30 m and 2 m above the lifting platform almost perpendicular to the plane of lifting. The Realanalyzer software automatically tracks the barbell during the lift in a video at 50 frames per second with an OpenCV template matching algorithm [13]. From the automatic tracking, the raw pixel data were smoothed with a cubic spline function. The smoothed pixel data represents the position data of the barbell. The barbell velocity and acceleration were computed as the 1^st^ and 2^nd^ derivative of the position data derived from the cubic spline function, respectively. Finally, real position, velocity, and acceleration data were computed via 2D image calibration (diameter of barbell plate). This system has previously shown good-to-excellent absolute (SEM), and relative (intraclass correlation coefficient [ICC]) test-retest reliability for the measurement of maximal vertical barbell acceleration (SEM = 0.18 m∙s^-2^, SEM% = 3.91%, ICC > 0.98), maximal vertical barbell velocity (SEM = 0.01 m∙s^-1^, SEM% = 0.72%, ICC > 0.99), and maximal vertical barbell lifting distance (SEM = 0.005 m, SEM% = 0.45%, ICC > 0.99) [14].

### Data processing

For ${\overset{-}{F}}_{\text{id}}$ and ${\overset{-}{F}}_{\text{we}}$, the acceleration phase (i.e., pull) of the snatch was extracted. The start of the lift was defined as the first video frame at barbell lift-off (vertical distance >0.225 m) and positive vertical barbell velocity. The end of the acceleration phase was set at the video frame corresponding to the maximal vertical barbell velocity. During the acceleration phase, the end of the 1^st^ pull and the end of the transition were classified by the lifter’s movement in accordance with barbell kinematics [15]. For the analysis of the individual vertical barbell acceleration time-series waveforms, all time-series data were temporally aligned (0–100%) using piecewise linear length normalization [16]. With this technique, subphases of the snatch lift (i.e., end of 1^st^ pull, end of transition) were aligned at a defined relative time point for all attempts to eliminate temporal differences of acceleration waveforms.

### Barbell force measurements during the acceleration phase of the snatch

#### Inverse dynamic approach

The instantaneous vertical barbell force (*F*_i_) at each video frame was computed from instantaneous vertical barbell acceleration (*a*_i_) starting at lift-off (i.e., vertical barbell velocity being null) until the end of the snatch acceleration phase (i.e., vertical barbell velocity being maximal), acceleration due to gravity (*g*) and barbell mass (*m*) according to Newton’s second law of motion as:

$$F_{i}\;=\;\left(a_{i}+g\right)\times m$$ (1)

The instantaneous vertical barbell acceleration during the acceleration phase (i.e., pull) of the snatch was computed as 2^nd^ derivative from video analysis using the software Realanalyzer. The mean vertical barbell force from vertical barbell acceleration (${\overset{-}{F}}_{\text{id}}$) was calculated as the average of *F*_i_ from lift-off until the end of the acceleration phase (i.e., maximal vertical barbell velocity), with *n* being the number of data points over the whole acceleration phase:

$${\overset{-}{F}}_{id}\;=\;\frac{1}{n}\sum_{i\;=\;1}^{n}F_{i}$$ (2)

#### Work-energy approach

During the acceleration phase of the snatch, muscles of the weightlifter produce mechanical work to elevate the barbell mass (*m*) from the floor at a height of 0.225 m (radius of barbell plates) until the lower limbs are completely extended while the arms are straight. This corresponds to the distance of acceleration until maximal vertical barbell velocity. This vertical acceleration distance (*h*_acc_) is highly individual and depends on the weightlifter’s anthropometric features. From this position, the barbell travels a further distance up (*h*_tr_) due to its maximal vertical velocity (*v*_max_) (i.e., projectile motion). According to Samozino et al. [11], the total work done (*W*_t_) during the acceleration phase is equal to the potential-energy change between lift-off and maximal height. Of note, during the snatch, weightlifters exert an additional force (denoted as “rest work”) on the barbell after reaching *v*_max_ (i.e., after the acceleration phase is completed) [17, 18]. Due to the weightlifters “rest work”, the total vertical travel distance of the barbell after *v*_max_ exceeds the theoretical travel distance achieved from projectile motion (*h*_tr_) [17, 18]. To accurately reflect the work that was produced during the acceleration phase, the sum of *h*_acc_ and *h*_tr_ was used to define the maximal barbell height for the calculation of *W*_t_:

$$W_{t}\;=\;m\times g\times \left(h_{acc}+h_{tr}\right)$$ (3)

Since the weightlifter’s lower limbs muscles produce *W*_t_, the amount of *W*_t_ at the barbell is equal to the product of *h*_acc_ multiplied by the mean vertical barbell force during the acceleration phase (${\overset{-}{F}}_{\text{we}}$). A rearrangement of this equation results in the following formula:

$${\overset{-}{F}}_{we}\;=\;\frac{W_{t}}{h_{acc}}$$ (4)

The inclusion of (Eq 3) in (Eq 4) provides (Eq 5) [11]:

$${\overset{-}{F}}_{we}\;=\;m\times g\left(\frac{h_{tr}}{h_{acc}}+1\right)$$ (5)

The vertical travel distance of the barbell from projectile motion (*h*_tr_) was calculated using maximal vertical barbell velocity:

$$h_{tr}\;=\;\frac{v_{max}{}^{2}}{2g}$$ (6)

Finally, *h*_acc_, *v*_max_, and the mass of the barbell were used as input parameters to compute ${\overset{-}{F}}_{\text{we}}$ (Eq 5), with *h*_acc_ and *v*_max_ being measured during the snatch from video analysis (i.e., Realanalyzer).

### Statistical analyses

The level of statistical significance for all tests was set at *p* ≤ 0.05. All statistical analyses were conducted using R (version 4.0.2). Normal distribution was assessed and confirmed using the Shapiro-Wilk test. The absence of heteroscedasticity (i.e., the measurement error is related to the magnitude of the measured variable) of the measurements was confirmed using the Breusch-Pagan test. Therefore, no log-transformation of the raw data was required. Descriptive statistics were presented as means and standard deviations (SD).

The mean difference between ${\overset{-}{F}}_{\text{id}}$ and ${\overset{-}{F}}_{\text{we}}$ was analyzed using a paired-sample *t*-test alongside effect size (*d*) and 95% confidence limits. The effect size was interpreted using the conventions outlined by Hopkins [19] as small (|*d*| > 0.2), moderate (|*d*| > 0.6), large (|*d*| > 1.2), very large (|*d*| > 2.0), or extremely large (|*d*| > 4.0). An effect size <0.2 was deemed trivial. The correlation between ${\overset{-}{F}}_{\text{id}}$ and ${\overset{-}{F}}_{\text{we}}$ was assessed using the Pearson product-moment correlation coefficient (*r*) with 95% confidence limits. Thresholds for the correlation coefficient were considered small (|*r*| > 0.1), medium (|*r*| > 0.3), large (|*r|* > 0.5), very large (|*r|* > 0.7), and extremely large (|*r|* > 0.9) [20]. The measurement error was computed as the standard deviation of the difference (SDD) scores (i.e., ${\overset{-}{F}}_{\text{id}}$ minus ${\overset{-}{F}}_{\text{we}}$) with 95% confidence limits. Additionally, a Bland-Altman analysis was used to compute the systematic bias (i.e., mean of measurements with 95% confidence limits) and 95% limits of agreement (systematic bias ± 1.96 × SDD) with 95% confidence limits. Significant systematic bias was given if the range of the 95% confidence limits of the mean differences did not contain the value 0. Also, a Deming regression was performed to test for constant and proportional bias between the approaches [21]. Significant constant bias was present if the range of the 95% confidence limits of the intercept did not contain the value 0 and significant proportional bias was present if the range of the 95% confidence limits of the slope did not contain the value 1 [22].

To analyze if the waveforms of the vertical barbell acceleration time-series data were associated with individual differences between ${\overset{-}{F}}_{\text{id}}$ and ${\overset{-}{F}}_{\text{we}}$ values, first, a principal component analysis of time-normalized acceleration waveforms was conducted. The principal component analysis is a data reduction technique that extracts dominant patterns (principal components, PC) of variability in the waveforms and reduces the entire data to a few PC (expressed as PC loadings and PC scores) that corresponds to the unique characteristics of the original waveforms [23]. The direction of variance in the waveforms is captured by the PC loadings. Of note, PC scores indicated the degree to which the shape of the individual waveform deviated from the average waveform. The principal component analysis is based on the singular value decomposition method with mean-centered acceleration time-series data. For this study, PC were retained that accounted for 90% of the cumulative explained variance [24]. To visualize the change in acceleration waveform associated with each PC, high and low PC acceleration waveforms (a_HL_) were created. For this purpose, the standard deviation of PC scores (SD_PCs_) was multiplied by the corresponding PC loadings (PCl_i_) and added or subtracted to the instantaneous average acceleration waveform of all attempts (${\overset{-}{a}}_{i}$) [25]:

$$a_{HL\_i}\;=\;{\overset{-}{a}}_{i}\pm SD_{PCs}\times PCl_{i}$$ (7)

Second, a forward stepwise multiple linear regression model on PC scores of the retained PC was used to analyze the linear relationship between the acceleration waveforms extracted by the principal component analysis and the observed differences in ${\overset{-}{F}}_{\text{id}}$ and ${\overset{-}{F}}_{\text{we}}$ values. Adjusted R^2^ values (R^2^_adj_) were used as selection criteria for the number of predictor variables (i.e., PC scores) of the multiple linear regression model.

## Results

### Concurrent validity

Results indicated a non-significant and trivial (*p* > 0.05, *d* = -0.04) mean difference between ${\overset{-}{F}}_{\text{id}}$ and ${\overset{-}{F}}_{\text{we}}$. The magnitude of the correlation coefficient between ${\overset{-}{F}}_{\text{id}}$ and ${\overset{-}{F}}_{\text{we}}$ was extremely large with *r* = 0.99 (Table 1).

**Table 1:** Comparison between the mean vertical barbell force computed from an inverse dynamic versus the work-energy approach during snatch performance.

${\overset{-}{F}}_{\text{id}}$ [N]	${\overset{-}{F}}_{\text{we}}$ [N]	diff [%]	*t*	*p*	*d* (95% CL)	*r* (95% CL)	SDD [N] (95% CL)	SDD% [%]
1974.9±307.7	1986.2±294.8	−0.57	−1.487	0.148	−0.04 (−0.08;0.01)	0.99 (0.98;1.00)	41.6 (33.1;55.9)	2.1

${\overset{-}{F}}_{\text{id}}$ = mean vertical barbell force computed from inverse dynamic approach (mean±SD), ${\overset{-}{F}}_{\text{we}}$ = mean vertical barbell force computed from work-energy approach (mean±SD), diff = mean percentage difference, *t* = *t*-score from the paired-sample *t*-test, *p* = *p*-value from the paired-sample *t*-test, *d* = effect size, *r* = Pearson product-moment correlation coefficient, SDD = standard deviation of differences, SDD% = percentage standard deviation of differences, 95% CL = 95% confidence limits.

**Fig 1:**
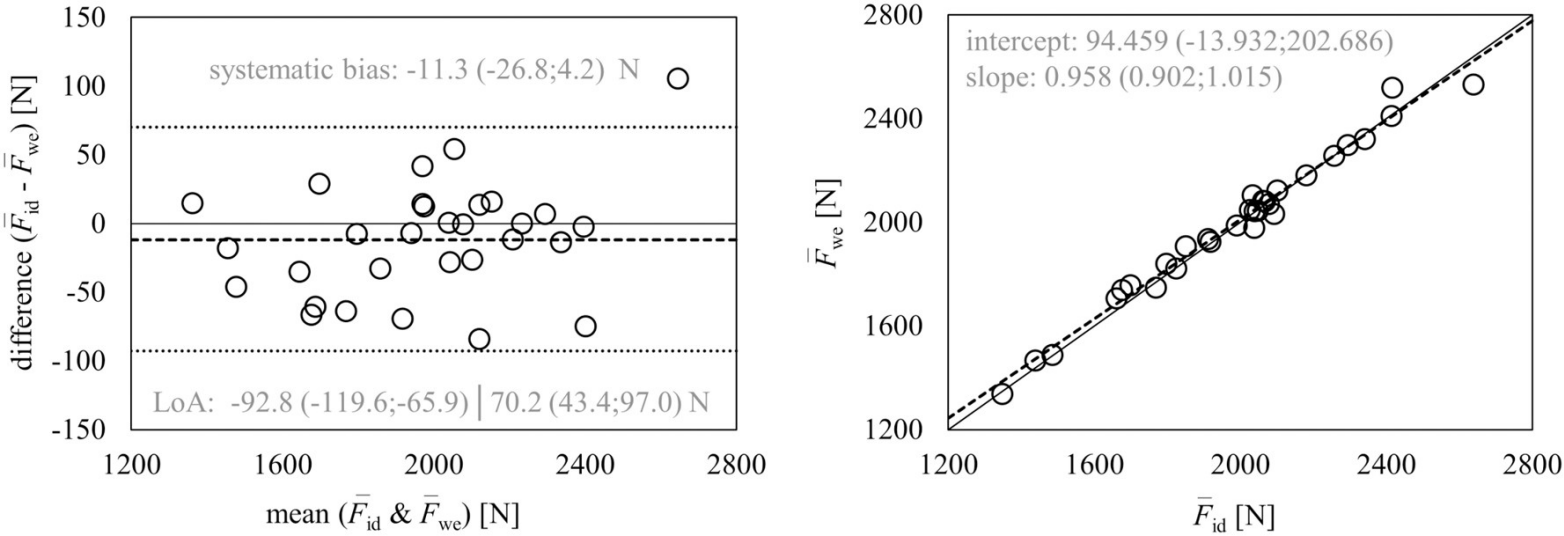
Results of the Bland-Altman analysis (left) and Deming regression (right) for the comparison between ${\overset{-}{F}}_{\text{id}}$ and ${\overset{-}{F}}_{\text{we}}$. Deming regression plot with the fitted linear model (dashed line) and the identity line (${\overset{-}{F}}_{\text{id}}\;=\;{\overset{-}{F}}_{\text{we}}$, slope = 1) (solid line). Bland-Altman plot with mean difference between methods (dashed line) and 95% limits of agreement (dotted lines). Regression parameters were reported as slope/intercept with 95% confidence limits, difference was reported as mean with 95% confidence limits, limits of agreement were reported as systematic bias ± 1.96 × SDD with 95% confidence limits. ${\overset{-}{F}}_{\text{id}}$ = mean vertical barbell force computed from inverse dynamic approach, ${\overset{-}{F}}_{\text{we}}$ = mean vertical barbell force computed from work-energy approach, LoA = limits of agreement.

The results of the Deming regression did not reveal a constant bias (i.e., confidence limits of intercept contained the value 0) or proportional bias (i.e., confidence limits of slope contained the value 1). The Bland-Altman analysis did not show any significant systematic bias (i.e., confidence limits of the mean differences contained the value 0) (Fig 1). The SDD was 41.6 N, and the 95% limits of agreement ranged between −92.8 N and 70.2 N.

### Barbell acceleration waveforms

**Fig 2:**
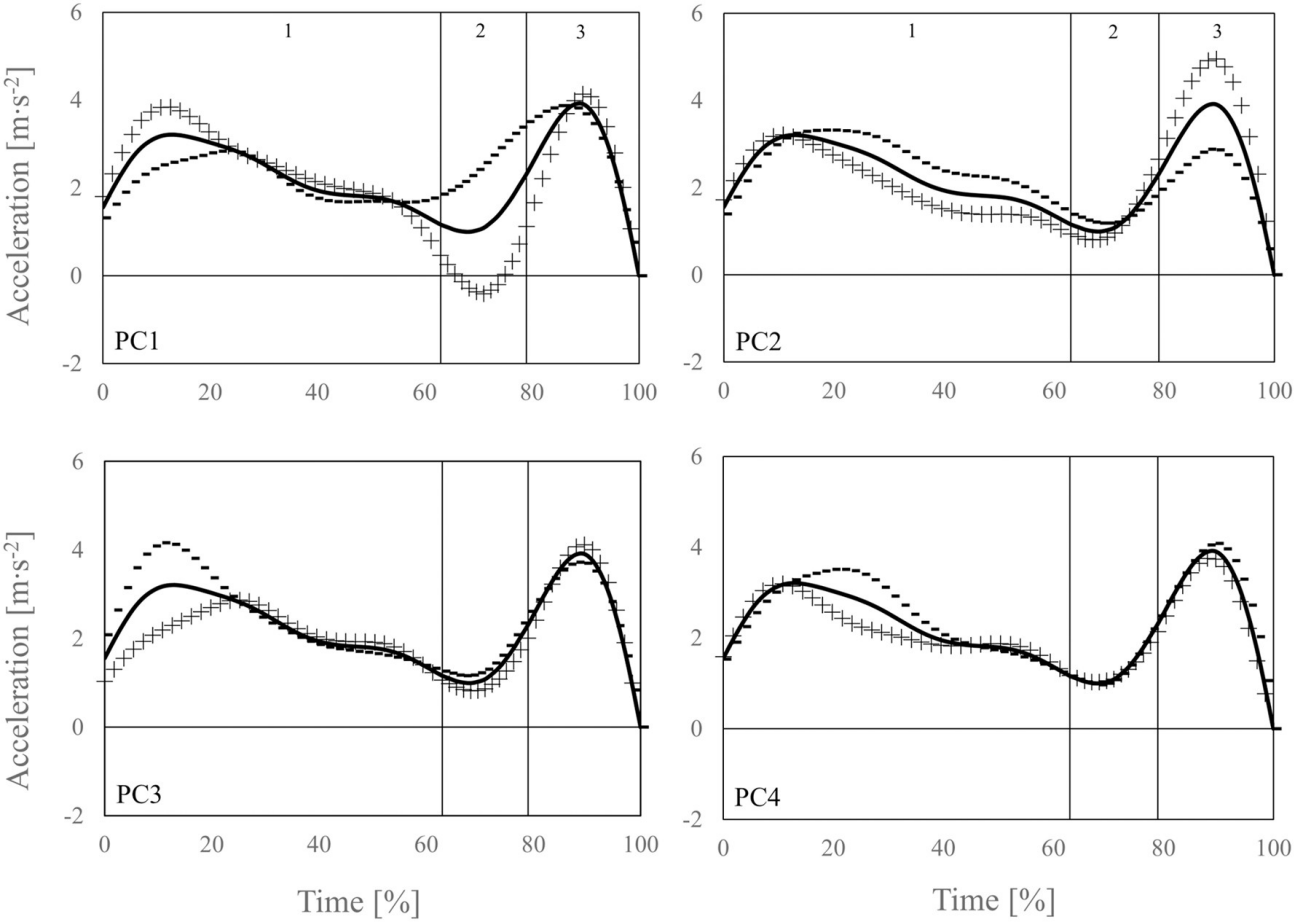
Vertical barbell acceleration data for the acceleration phase (0–100%) of the snatch with the effect of high (+ signs) and low (− signs) PC scores for PC1-4 on the average acceleration waveform (black solid lines). The numbers 1–3 in the upper graphs separate the 1^st^ pull, from transition, and 2^nd^ pull during the acceleration phase.

Four PC were extracted by the principal component analysis explaining 89.9% of the variance in the vertical barbell acceleration waveforms (PC1 = 47.4%, PC2 = 22.3%, PC3 = 14.8%, and PC4 = 5.4%). Regarding the average vertical barbell acceleration profile, the PC1 measured the variation within the waveforms at the beginning of the 1^st^ pull and during the transition, the PC2 during the 1^st^ pull and 2^nd^ pull, and the PC3 and PC4 at the beginning of the 1^st^ pull (Fig 2).

The stepwise multiple linear regression using PC scores of all retained PC revealed a model containing the PC scores of PC1, PC2, and PC3 as variables to predict differences of ${\overset{-}{F}}_{\text{id}}$ and ${\overset{-}{F}}_{\text{we}}$ values (Table 2).

**Table 2:** A multiple linear regression model to predict differences in vertical barbell force during the snatch computed from the inverse dynamic approach and the work-energy approach using PC scores of principal component analysis from vertical barbell acceleration waveforms.

R^2^_adj_	SEE [N]	Coefficients (95% CL)			
		Intercept	PC1 scores	PC2 scores	PC3 scores
0.86	15.8	−11.29 (−17.20;−5.37)	4.81 (3.67;5.94)	−6.66 (−8.33;−4.99)	−5.69 (−7.72;-3.66)

R^2^_adj_ = adjusted R^2^, SEE = standard error of estimate, PC = principal components, 95% CL = 95% confidence limits.

## Discussion

This study aimed to examine the accuracy of ${\overset{-}{F}}_{\text{we}}$ in comparison with ${\overset{-}{F}}_{\text{id}}$ during the snatch exercise in elite male weightlifters who competed at the World Championships 2018. Concurring with the hypothesis, ${\overset{-}{F}}_{\text{we}}$ was not significantly different from ${\overset{-}{F}}_{\text{id}}$ and the two measures correlated with an extremely large magnitude. These findings indicate the overall high agreement between the inverse dynamic and the work-energy approach to compute the mean vertical barbell force during the acceleration phase of the snatch in male elite weightlifters.

For the squat jump push-off, Samozino et al. [11] used the work-energy approach to compute the mean ground reaction force in physically active male participants aged 27 years. These authors reported ${\overset{-}{F}}_{\text{we}}$ to be valid in agreement with the “direct” measurement of mean ground reaction force using a force plate (Bland-Altman analysis: systematic bias of −11.5±25.4 N, limits of agreement −61.3 to 38.3 N). The percentage of measurement error (SDD% = 2.1%) of the current study is very similar to the error reported by Samozino et al. [11] (SDD% = 2.0%). In addition, Rahmani et al. [12] used the work-energy approach to compute the mean vertical system (barbell plus upper limbs) force during the guided bench press throw in trained male individuals aged 28 years. They demonstrated that ${\overset{-}{F}}_{\text{we}}$ is valid and in agreement with ${\overset{-}{F}}_{\text{id}}$ that was computed from an accelerometer attached to the barbell [12]. Unfortunately, Rahmani et al. [12] did not use Bland-Altman analysis to assess the measurement error; instead they reported mean differences only. Although not in terms of vertical barbell force, a subjective (no statistical evaluation) high agreement between the work-energy and the inverse dynamic approach was reported to compute a vertical performance index for weightlifting [26]. Recently, Sandau, Chaabene and Granacher [27] supported the reliability of the work-energy approach using video analysis to compute mean vertical barbell force (i.e., F_0_ of force-velocity relationship) during the snatch pull (SEM% = 1.09%) in male elite weightlifters aged 28 years.

It has previously been reported that the acceleration time-series from video-analysis is often erroneous due to the increased noise content during mathematical differentiation of displacement data [8]. Although the mean difference between both computational approaches was not statistically significant, the limits of agreement displayed a high variability ranging from −92.8 N to 70.2 N, representing a total error of 8.2% (163.0 N). This individual differences can be attributed to the error-prone vertical barbell acceleration measured by video analysis that was used in the inverse dynamic approach (i.e., reference method). This assumption is strengthened by the current findings from the multiple linear regression analysis using PC scores. In fact, the multiple linear regression model explained 86% of the variance in the individual differences between ${\overset{-}{F}}_{\text{we}}$ and ${\overset{-}{F}}_{\text{id}}$ when predicted with PC scores of PC1, PC2, and PC3 (i.e., pattern of variability in the waveforms of vertical barbell acceleration time-series data). In general, for each PC, athletes with lower PC scores follow the extracted waveform pattern that was denoted by minus (−) signs and athletes with higher PC scores follow the waveforms that were denoted by plus (+) signs (Fig 2). As indicated through the multiple linear regression model, scores of PC1 were positively correlated with the difference between ${\overset{-}{F}}_{\text{we}}$ and ${\overset{-}{F}}_{\text{id}}$ (i.e., higher PC1 scores = positive difference, lower PC1 scores = negative difference), while scores of PC2 and PC3 were negatively correlated (i.e., higher PC2/3 scores = negative difference, lower PC2/3 scores = positive difference). In addition, based on the regression coefficients, scores of PC2 and PC3 have a larger effect on the differences between ${\overset{-}{F}}_{\text{we}}$ and ${\overset{-}{F}}_{\text{id}}$ than scores of PC1. Transferring this weighting on the waveform level, it can be concluded that the variation in waveforms during the 1^st^ pull (PC3) and 2^nd^ pull (PC2) has a larger effect on the measured differences between the computational methods than waveforms during the transition (PC1).

To sum up, individual differences between ${\overset{-}{F}}_{\text{id}}$ and ${\overset{-}{F}}_{\text{we}}$ can be predicted from vertical barbell acceleration waveforms, making the supposed “random error” explainable (i.e., systematic). Given that individual differences can be attributed to the error-prone barbell acceleration that is used within the inverse dynamic approach, we recommend to use the work-energy approach to measure mean vertical barbell force.

This study is not without limitations. First, a 50 Hz video analysis was used. Earlier studies analyzing barbell acceleration (marker-based tracking) used a sampling frequency >200 Hz, which could provide more accurate measures [6, 28]. However, results of spectral analysis (i.e., detecting frequency bandwidth) demonstrated that a sampling rate of at least 25 Hz is adequate to measure movement velocity during resistance training exercises (i.e., squat jump, countermovement jump, squat, bench press) with an error less than 0.1% using a position encoder [29]. Additionally, Winter [10] reported that segment acceleration during walking can adequately be measured using 25 Hz video analysis. Second, the vertical barbell force did not take the force to accelerate the lifter’s body and the horizontal force into account. Therefore, the mean vertical barbell force did not reflect the entire muscular effort. However, the overall horizontal force exerted by a weightlifter during a lift is much smaller compared with the vertical force [30]. Furthermore, transferring a proper vertical force (acceleration) on the barbell is the final goal during a lift that needs to be improved by training to increase weightlifting performance [28, 31]. These facts need to be considered when computing the mean vertical barbell force.

## Conclusions

On average, the results of this study showed similar mean vertical barbell force outcomes between the inverse dynamic and work-energy approaches during the snatch using video analysis. However, because the inverse dynamic approach could be biased due to the vertical barbell acceleration, especially during extreme acceleration waveforms, the work-energy approach appears to represent a more accurate procedure to calculate mean vertical barbell force. Given the comparable biomechanical characteristics during the acceleration phase (i.e., pull) of weightlifting derivatives, the work-energy approach can also be applied with other weightlifting exercises (e.g., snatch pull, clean, clean pull) to measure mean vertical barbell force during the acceleration phase.

## Supporting information

S1 Table Sample and kinematic measures data. (PDF)

S2 Table Vertical barbell acceleration time-series data. (PDF)
