# Supplementary material for: Concurrent validity of barbell force measured from video-based barbell kinematics during the snatch in male elite weightlifters
Source: PLoS One. 2021 Jul 19;16(7):e0254705. doi: 10.1371/journal.pone.0254705 (PMC8289080; doi:10.1371/journal.pone.0254705)
Supplement: S1 Table — (PDF) [file pone.0254705.s001.pdf]

bodyweight category	age [y]	athlete number	barbell mass [kg]	vmax [m/s]	hacc [m]	Fid [N]	Fwe [N]
55	27	1	120	1.73	0.62	1446.50	1464.60
55	26	2	120	1.81	0.61	1454.69	1500.87
55	23	3	113	1.79	0.74	1368.95	1354.13
61	29	4	137	1.85	0.63	1656.61	1717.08
61	25	5	133	1.85	0.64	1627.03	1662.23
61	24	6	135	1.90	0.64	1643.25	1709.63
67	25	7	145	1.90	0.69	1736.08	1799.73
67	26	8	152	2.05	0.69	1882.71	1951.94
67	22	9	144	1.67	0.75	1711.46	1682.41
73	25	10	158	1.95	0.72	1980.43	1968.07
73	25	11	151	1.93	0.72	1842.26	1874.94
73	21	12	150	1.85	0.78	1792.74	1800.41
81	34	13	172	1.87	0.71	2087.93	2114.25
81	29	14	165	1.76	0.74	1977.27	1962.98
81	20	15	163	2.12	0.80	2028.79	2056.88
89	24	16	166	1.65	0.72	1937.06	1943.87
89	25	17	169	1.89	0.79	2039.41	2038.76
89	20	18	163	1.92	0.86	1991.02	1949.19
96	30	19	186	2.03	0.74	2329.23	2342.71
96	24	20	181	1.97	0.77	2232.77	2232.80
96	25	21	175	1.87	0.85	2077.09	2077.70
102	27	22	177	1.67	0.85	2080.84	2026.53
102	28	23	176	1.90	0.82	2126.85	2113.28
102	21	24	175	1.95	0.75	2078.06	2161.97
109	21	25	190	1.88	0.79	2297.19	2290.23
109	27	26	186	1.89	0.85	2202.86	2214.42
109	28	27	179	1.91	0.84	2160.90	2145.03
+109	25	28	217	2.03	0.97	2697.96	2592.56
+109	24	29	200	1.95	0.79	2364.01	2438.91
+109	25	30	199	1.94	0.84	2394.62	2396.99
