# Supplementary material for: Concurrent validity of barbell force measured from video-based barbell kinematics during the snatch in male elite weightlifters
Source: PLoS One. 2021 Jul 19;16(7):e0254705. doi: 10.1371/journal.pone.0254705 (PMC8289080; doi:10.1371/journal.pone.0254705)
Supplement: S2 Table — (PDF) [file pone.0254705.s002.pdf]

video frame number	vertical barbell acceleration (lift-off to vmax) [m/s^2]																													
	athlete 1	athlete 2	athlete 3	athlete 4	athlete 5	athlete 6	athlete 7	athlete 8	athlete 9	athlete 10	athlete 11	athlete 12	athlete 13	athlete 14	athlete 15	athlete 16	athlete 17	athlete 18	athlete 19	athlete 20	athlete 21	athlete 22	athlete 23	athlete 24	athlete 25	athlete 26	athlete 27	athlete 28	athlete 29	athlete 30
1	1.5515	1.1791	1.7322	0.984	0.7557	1.269	0.3553	2.6738	1.7626	1.5422	1.5931	1.0105	2.2124	1.6864	2.3128	0.4688	1.4498	1.8744	1.638	1.9019	1.6061	3.1515	2.8883	0.9134	1.7871	0.6911	0.1546	1.7869	1.5395	2.2722
2	2.281	1.6645	2.3747	1.4105	0.9856	1.6636	0.5372	3.4799	2.473	2.0249	2.18	1.5065	2.9133	2.3181	2.9636	0.7761	1.9917	2.7559	2.2879	2.7193	2.2514	3.9451	3.9399	0.8565	2.5695	0.9265	0.2392	2.7636	2.0272	3.3344
3	2.9753	2.0849	2.8723	1.8146	1.1934	1.9693	0.7369	3.9185	3.0782	2.5038	2.6573	2.0081	3.4925	2.8238	3.4152	1.1597	2.4541	3.5008	2.8558	3.4783	2.8094	4.4246	4.7196	0.6419	3.2735	1.2178	0.3718	3.7712	2.3791	4.2614
4	3.5204	2.3693	3.1268	2.1381	1.4114	2.1697	0.9578	3.9038	3.4837	2.9977	2.9551	2.4522	3.8501	3.1161	3.6429	1.591	2.7839	3.9746	3.3135	4.0605	3.1874	4.5605	5.1239	0.3229	3.7826	1.5551	0.6045	4.6725	2.5519	4.8736
5	3.8265	2.493	3.1242	2.3357	1.6622	2.2959	1.2121	3.5353	3.6672	3.4877	3.0669	2.7847	3.9693	3.1802	3.6777	2.0485	2.9774	4.117	3.6483	4.3766	3.3395	4.4042	5.1573	-0.0264	4.0347	1.9273	0.9566	5.3465	2.5735	5.0662
6	3.852	2.4866	2.9448	2.3885	1.935	2.3957	1.505	3.0496	3.6821	3.9102	3.041	2.9741	3.9056	3.0736	3.5708	2.5118	3.0688	3.9678	3.8592	4.4069	3.2762	4.0581	4.8995	-0.3233	4.0358	2.3231	1.3959	5.7151	2.5158	4.8553
7	3.618	2.4153	2.7227	2.3169	2.1888	2.4951	1.819	2.6889	3.6172	4.1871	2.9439	3.0197	3.7329	2.8866	3.3723	2.9498	3.1009	3.6509	3.9532	4.2088	3.0546	3.6366	4.4508	-0.4781	3.8434	2.7243	1.8505	5.7591	2.4356	4.3684
8	3.206	2.3373	2.5767	2.1799	2.376	2.5836	2.1201	2.5696	3.5408	4.2737	2.8267	2.9472	3.4986	2.6931	3.1284	3.3185	3.1022	3.3212	3.9368	3.8879	2.758	3.2354	3.8965	-0.3966	3.5371	3.1034	2.2401	5.524	2.3359	3.778
9	2.7283	2.2686	2.5568	2.049	2.4617	2.6294	2.375	2.6384	3.4685	4.1892	2.7155	2.7966	3.2238	2.53	2.88	3.573	3.0831	3.099	3.8133	3.5543	2.4703	2.9156	3.3056	0.0036	3.196	3.4276	2.5078	5.1103	2.1831	3.2242
10	2.2863	2.1848	2.6364	1.972	2.4356	2.602	2.5625	2.735	3.3748	3.999	2.6161	2.6137	2.932	2.4059	2.6511	3.686	3.0454	3.0277	3.5879	3.2843	2.2575	2.7035	2.7383	0.7565	2.8868	3.6663	2.6442	4.6404	1.958	2.7738
11	1.941	2.0531	2.7494	1.951	2.3188	2.4852	2.6759	2.7044	3.2261	3.7645	2.5204	2.4437	2.6631	2.3165	2.4476	3.6576	2.984	3.074	3.2784	3.098	2.1595	2.5983	2.2397	1.8023	2.6534	3.7964	2.6946	4.214	1.6876	2.4299
12	1.7066	1.8672	2.8379	1.947	2.1587	2.2849	2.7243	2.4758	3.0071	3.5069	2.4157	2.3195	2.4538	2.2529	2.2696	3.5056	2.8809	3.1611	2.9272	2.9633	2.1824	2.5795	1.8284	2.9728	2.5066	3.8037	2.7368	3.8782	1.4326	2.1671
13	1.5628	1.6578	2.8814	1.912	2.0061	2.0374	2.7293	2.0709	2.729	3.2169	2.2998	2.2522	2.3054	2.2038	2.1231	3.2475	2.7108	3.2138	2.6028	2.827	2.2915	2.6068	1.4975	4.0318	2.4217	3.6871	2.8378	3.6275	1.2488	1.9599
14	1.4767	1.481	2.8973	1.8215	1.8918	1.8068	2.7132	1.5651	2.4302	2.8917	2.1898	2.2313	2.1792	2.1623	2.0178	2.8943	2.4604	3.1918	2.3776	2.6486	2.4236	2.6214	1.2281	4.7588	2.352	3.4621	3.0158	3.4257	1.1605	1.7972
15	1.4219	1.3943	2.9185	1.6825	1.8228	1.6578	2.6907	1.048	2.1724	2.5553	2.1127	2.2323	2.0253	2.1383	1.955	2.4629	2.1517	3.1007	2.29	2.4148	2.5177	2.5648	1.0086	5.0298	2.2556	3.1579	3.2281	3.2395	1.1618	1.6828
16	1.3868	1.4292	2.9619	1.5154	1.7934	1.6199	2.6716	0.6033	2.0174	2.2448	2.0872	2.2245	1.8133	2.1568	1.9166	1.9879	1.8425	2.9827	2.3141	2.132	2.5381	2.4096	0.8462	4.8529	2.1157	2.8097	3.3947	3.0621	1.2337	1.6211
17	1.3729	1.568	3.0099	1.3358	1.793	1.6734	2.6657	0.2992	1.99	1.9824	2.1128	2.1782	1.5413	2.2359	1.8689	1.5207	1.5993	2.8898	2.3677	1.8099	2.4753	2.1755	0.7623	4.3473	1.941	2.4581	3.4452	2.9122	1.3546	1.5967
18	1.3851	1.7451	3.0202	1.1583	1.8029	1.7668	2.6799	0.1712	2.0574	1.7696	2.1724	2.0786	1.226	2.3624	1.7784	1.1182	1.4618	2.8497	2.3668	1.4547	2.3399	1.9162	0.7715	3.6877	1.7536	2.151	3.3561	2.8105	1.4996	1.5728
19	1.4195	1.8777	2.9511	1.0109	1.7994	1.8513	2.7129	0.2057	2.145	1.6063	2.2465	1.942	0.8854	2.4904	1.6357	0.8266	1.4296	2.8444	2.2841	1.0764	2.1561	1.6932	0.8641	3.0395	1.5796	1.9299	3.153	2.7597	1.6392	1.5129
20	1.4632	1.9127	2.7846	0.9287	1.7651	1.9023	2.7578	0.3473	2.1769	1.5011	2.3279	1.815	0.5299	2.5667	1.4722	0.6631	1.4783	2.8192	2.16	0.7027	1.9569	1.5516	1.0048	2.5094	1.442	1.8043	2.8874	2.7411	1.7452	1.3983
21	1.5035	1.857	2.5353	0.9256	1.6924	1.9165	2.8036	0.5293	2.1086	1.4583	2.4204	1.746	0.1718	2.5577	1.3512	0.611	1.585	2.7154	2.062	0.3905	1.7772	1.5037	1.1473	2.13	1.3527	1.7384	2.6175	2.7201	1.7997	1.2215
22	1.537	1.774	2.245	0.9808	1.5802	1.9024	2.8298	0.7125	1.9307	1.464	2.5174	1.7468	-0.1511	2.4532	1.3308	0.632	1.7332	2.5065	2.0345	0.2257	1.6482	1.5225	1.2522	1.8801	1.3016	1.6719	2.3964	2.6485	1.8041	0.9702
23	1.5693	1.7541	1.9632	1.0539	1.4401	1.8751	2.8062	0.9068	1.6529	1.4936	2.5865	1.7792	-0.3616	2.2517	1.4277	0.6891	1.8957	2.2116	2.0724	0.2998	1.5907	1.5496	1.2933	1.7198	1.2488	1.5564	2.2615	2.4718	1.7851	0.6265
24	1.6084	1.8778	1.7293	1.1106	1.308	1.8565	2.7077	1.1614	1.2912	1.5387	2.5865	1.7792	-0.3615	1.9445	1.6184	0.7646	2.0285	1.878	2.1282	0.6568	1.6069	1.5156	1.2553	1.6175	1.133	1.3764	2.228	2.1498	1.7876	0.1806
25	1.6558	2.1811	1.5677	1.1387	1.2364	1.8819	2.5314	1.5274	0.8639	1.6256	2.5096	1.702	-0.0763	1.5191	1.8676	0.8588	2.0913	1.5478	2.15	1.2435	1.6714	1.3612	1.1376	1.5586	0.909	1.1396	2.29	1.6787	1.8465	-0.3576
26	1.707	2.635	1.491	1.1512	1.2701	2	2.3006	2.0242	0.4018	1.8064	2.4061	1.5484	0.5056	0.9839	2.1477	0.9779	2.0727	1.2402	2.1277	1.9144	1.7326	1.0535	0.9651	1.5395	0.5898	0.857	2.4244	1.1011	1.9495	-0.947
27	1.7695	3.1535	1.5038	1.1824	1.4248	2.2554	2.0523	2.6274	-0.0351	2.1213	2.3601	1.353	1.3218	0.3962	2.4383	1.1264	1.9956	0.9665	2.1075	2.502	1.726	0.5982	0.7941	1.5571	0.2601	0.541	2.5894	0.5022	2.0213	-1.5035
28	1.8859	3.6306	1.6029	1.2813	1.6866	2.6609	1.8208	3.2838	-0.3491	2.5592	2.4382	1.1511	2.2534	-0.1339	2.7216	1.2976	1.9046	0.7587	2.1595	2.9033	1.5909	0.0435	0.7028	1.5993	0.0508	0.2149	2.7245	-0.0043	1.9512	-1.9018
29	2.1297	3.9849	1.7708	1.4974	2.0266	3.1847	1.6304	3.9364	-0.425	3.0473	2.6519	0.9594	3.1633	-0.4707	2.9815	1.4606	1.8506	0.6716	2.3371	3.1165	1.2867	-0.5253	0.7689	1.6444	0.0907	-0.0851	2.761	-0.3015	1.6492	-1.9972
30	2.5563	4.189	1.9716	1.859	2.4154	3.7585	1.5004	4.5441	-0.1747	3.4793	2.9502	0.7841	3.9375	-0.4972	3.2072	1.5598	1.8786	0.751	2.6602	3.2196	0.8127	-0.9948	1.0427	1.676	0.4548	-0.3145	2.6485	-0.3036	1.0947	-1.6655
31	3.136	4.266	2.1598	2.3618	2.8326	4.2914	1.4555	5.0825	0.4185	3.7558	3.2276	0.6437	4.5097	-0.1493	3.4012	1.5419	2.0184	1.0009	3.0997	3.3181	0.2271	-1.2397	1.531	1.7033	1.1279	-0.4275	2.3779	0.0157	0.3529	-0.8471
32	3.7196	4.2493	2.2974	2.9745	3.269	4.6797	1.5296	5.5183	1.2768	3.7982	3.3517	0.579	4.8524	0.5663	3.5775	1.3929	2.2766	1.371	3.5572	3.4822	-0.3401	-1.1504	2.1948	1.7591	2.0015	-0.3887	1.9823	0.6009	-0.4407	0.4227
33	4.0703	4.1254	2.356	3.6442	3.7144	4.8184	1.7569	5.775	2.2271	3.5331	3.2109	0.6427	4.9469	1.5602	3.7356	1.1619	2.6272	1.7592	3.8617	3.6877	-0.6948	-0.6722	2.9497	1.8715	2.9088	-0.183	1.5225	1.3029	-1.1173	2.0125
34	3.9565	3.8065	2.2988	4.2901	4.136	4.6211	2.1493	5.7204	3.0296	2.8851	2.7656	0.8773	4.7599	2.6511	3.8236	0.956	3.0048	2.0333	3.8072	3.7949	-0.6253	0.1549	3.667	2.0363	3.6749	0.1829	1.0766	1.8995	-1.5164	3.7104
35	3.2532	3.1644	2.0627	4.8046	4.4622	4.0409	2.668	5.2034	3.4401	1.8088	2.0669	1.2977	4.2502	3.5779	3.7256	0.9083	3.3109	2.0744	3.2225	3.5951	0.0018	1.1727	4.1878	2.2203	4.1464	0.6875	0.7378	2.1666	-1.5174	5.2469
36	1.993	2.1068	1.57	5.0773	4.5904	3.0822	3.2093	4.1302	3.2916	0	0	1.8787	3.4007	4.0543	3.2981	1.127	3.4335	1.8197	2.0462	2.9141	1.1437	2.1187	4.3539	2.3913	4.2011	1.3022	0.6044	1.9673	-1.0567	6.338
37	0	0.6459	0.7684	5.0256	4.4112	1.7982	3.6152	2.5313	2.5537			2.5456	2.244	3.8632	2.4405	1.6404	3.275	1.2689	0	1.7084	2.5555	2.6939	4.0469	2.5426	3.7711	1.9946	0.7557	1.2983	-0.1359	6.7597
38		0	0	4.6041	3.8417	0	3.7062	0	1.3289			3.1693	0	2.9457	1.1558	2.3618	2.7777	0.4518		0	3.8701	2.6685	3.219	2.6959	2.8804	2.7221	1.2174	0	1.1742	6.425
39				3.793	2.8609		3.3331		0			3.5851		1.4276	0	3.0952	1.9378	0			4.7349	1.9695	1.9085	2.8836	1.6512	3.4195	1.929		2.7346	5.4052
40				2.5961	1.5285		2.4371					3.6426		0		3.5866	0.8048				4.9279	0	0	3.1191	0.2547	3.9941	2.7335		4.3495	3.8785
41				1.0577	0		1.0833					3.2582				3.6132	0				4.4017			3.3674	0	4.3436	3.4017		5.7825	2.0492
42				0			0					2.4291				3.0708					3.2517			3.5301		4.3889	3.6915		6.7847	0
43												1.2074				2.006					1.6504			3.4611		4.0979	3.4298		7.1414	
44												0				0					0			3.0204		3.4873	2.5785		6.7273	
45																								2.1446		2.601	1.2428		5.5451	
46																								0		1.4862	0		3.7305	
47																										0			0	
